# Postpartum depression and associated factors among mothers in Bahir Dar Town, Northwest Ethiopia

**DOI:** 10.1186/s12991-019-0244-4

**Published:** 2019-09-10

**Authors:** Amsale Abebe, Getachew Tesfaw, Haregewoine Mulat, Getahun Hibdye, kalkidan Yohannes

**Affiliations:** 1 Department of Psychiatry, Felege Hiwot Referral Hospital, Bahir Dar University, Bahar Dar, Ethiopia; 2 Department of Psychiatry, College of Medicine and Health Sciences, University of Gondar, Gondar, Ethiopia; 3 Research and Training Department, Amanuel Mental Specialized Hospital, Addis Ababa, Ethiopia; 4 Department of Psychiatry, College of Medicine and Health Sciences, Dilla University, 419, Dilla, Ethiopia

**Keywords:** Postpartum depressive symptoms, Mothers

## Abstract

**Background:**

Postpartum depressive symptoms are the occurrence of major depressive episode within 4 weeks following delivery. Globally, 10%–20% of mothers suffer from depressive symptoms during their postpartum course. Therefore, assessing postpartum depressive symptoms has a pivotal role in ensuring that their mental health needs are met.

**Methods:**

An institution-based cross-sectional study was conducted including 511 mothers coming for postnatal care service in public health centers in Bahir Dar Town. Data were collected using a pre-tested, structured, and interviewer-administered questionnaire, while the Edinburgh Postnatal Depression Scale (EPDS) was used to assess individuals’ depressive symptoms. The systematic random sampling technique was employed to recruit the study participants. Adjusted odds ratio with a 95% confidence interval (CI) was used to declare the statistical significance of the factors.

**Results:**

Postpartum depressive symptoms among mothers were found to be 22.1 (95%, CI 18.6%, 25.8%). In multivariate logistic regression, stressful life events (AOR = 4.46, 95% CI 2.64, 7.54), domestic decision making (AOR = 4.26, 95% CI 2.54, 7.14), unplanned pregnancy (AOR = 1.86, 95% CI 1.02, 3.41), partner violence (AOR = 3.16, 95% CI 1.76, 5.67), and hospitalization of their babies (AOR = 2.24, 95% CI 1.17, 4.310) were factors significantly associated with postpartum depressive symptoms.

**Conclusions:**

Postpartum depressive symptoms among mothers were common in the study area. Stressful life events, lack of empowerment in domestic decision making, intimate partner violence, unplanned pregnancy, and hospitalization of their baby were factors significantly associated with postpartum depression. The Ministry of Health needs to give training on how to screen postpartum depressive symptoms among mothers, and interventions that would address the above factors would benefit in tackling further complications.

## Introduction

Depression is one of the most frequent psychiatric conditions among reproductive-age women [1]. It is the leading contributor to disease burden among childbearing women with depressive episode within 4 weeks following childbirth [2]. The World Health Organization stated that 20–40% of women in developing countries experienced depressive symptoms during pregnancy or after childbirth [3]. Globally, 10%–20% of mothers suffer from depressive symptoms during their postpartum period [4]. Half of the depressed women were expected to have an episode of depression following pregnancy [5]. In Ethiopia, depressive symptoms affect at least 1 in 20 following delivery [6]. Even though every woman is potentially at risk of developing postpartum depressive symptoms, an early age of childbearing is more vulnerable to develop postpartum depression [7]; low education level, poverty [8], poor social support [9], and childbirth without the presence of relatives [10] were factors that increased the risks of depressive symptoms after delivery. Besides the above factors, previous history of depression [11], obstetric complication, miscarriage [12], parous women, an unplanned pregnancy [13], intimate partner violence [14], stressful life events [15], and poor woman autonomy [16] were factors of postpartum depressive symptoms. Postpartum depressive symptoms have physical and mental health consequences for mothers and their families. Postpartum depression is an extreme feeling of sadness, hopelessness, the recurrent thought of death, and risk of the mother harming herself [14, 17]. Depressed mothers provide inadequate care to their children, show negative parenting behavior, and present poor mother–infant bonding [18]. Children of depressed mothers had impaired emotional, social, and cognitive development [19–21].

Women’s health is a public concern, but the diagnosis of postpartum depressive symptoms has received little attention. Although nearly 90% of the world’s children live in low- and middle-income countries, we know little about the prevalence of maternal depressive symptoms in Ethiopia. Therefore, the finding of this study will fill the gap in making a necessary solution by assessing the prevalence and associated factors of postpartum depression among mothers attending maternal and child health clinics in the study area.

## Methods and materials

### Study setting and study design

An institution based cross-sectional study was conducted from May to June 2018 at public health centers in Bahir Dar Town, Northwest Ethiopia.

### Study population

All women attending postnatal care service clinics and gave birth within 6 months of postpartum were included. Women less than 18 years of age who gave birth 2 weeks prior to the data collection period and critically ill patients were excluded.

### Sampling procedures

The sample size was calculated by using the single population proportion formula involving the use of Epi-Info version 7 with a 95% CI, a 5% margin of error, and taking the prevalence of postpartum depressive symptoms as 32.8% [22]. Assuming a 5% non-response rate, 555 women were recruited randomly using the systematic sampling technique. The sampling interval was determined by dividing the total study population who had follow-up during the data collection period by the sample size, and then the starting point was randomly selected.

### Data collection

Data were collected using a pre-tested interviewer-administered questionnaire, which contained socio-demographic characteristics (age, education, occupation, marital status). Abuse assessment screening tool had three questions to detect the prevalence of sexual or physical abuse during pregnancy and was sensitive to screen abuse [23]. Women’s autonomy screening had three components: freedom of movement, decision making regarding children, and decision making regarding household tasks. The Stressful Life Events Screening Questionnaire (SLESQ) had a 13-item self-reporting questionnaire developed as a general traumatic event that assesses lifetime exposure to traumatic events and has been adapted for use as a basis for yes or no questions [24]. It was developed for use with community samples, and the psychometric properties are being tested in samples of low-income countries [25, 26].

Social support was collected by the Oslo 3-item social support scale which has a 3-item questionnaire commonly used to assess social support and used in several studies. The sum score scale ranges from 3 to 14, and has three broad categories: “poor support” 3–8, “moderate support” 9–11, and “strong support” 12–14 [27]. It has been used in Ethiopia in various settings including the community for this study [28, 29].

Edinburgh Postnatal Depression Scale (EPDS) has ten inventory questions that investigates feelings that occurred during the previous 7 days and each question had four answers rated from 0 to 3 with the cutoff score ≥ 13. It was validated as a screening tool to detect postnatal depressive symptoms in Ethiopia and found to have sensitivity of 78.9% and specificity of 75.3% [30, 31].

### Data processing and analysis

Data were entered into Epi Info software after checking completeness and imported to SPSS version 21 for analysis. The bivariable and multivariable logistic regression analyses were done to see the association of each independent variable with the outcome variable. Variables with a *P* value less than 0.2 were considered as the multivariate logistic regression model to identify the effect of each independent variable with the outcome variables. The strength of the association was evaluated using the adjusted odds ratio with a 95% CI, and a *P* value of less than 0.05 was considered statistically significant.

## Results

### Socioeconomic and demographic characteristics

A total of 511 participants were included in the study with the response rate of 97.3%. The mean age of the respondents was 24.3 (± 3.83) years. Out of the participants, 353 (69.1%) were of age of 20–29 years and 483 (94.5%) were married. Nearly 3 out of 5 of the 305 (59.7%) mothers were unemployed and almost all 485 (94.9%) of the women had formal education. Pertaining to income, for nearly 2 out of 5 of the 198 (38.7%) respondents, the average monthly income of the family was between 1201 and 2500 birr and the mean was 2856 ETB (Table 1).

**Table 1:** Socio-demographic characteristics of mothers attending maternal and child health clinics of public health centers at Bahir Dar, Northwest Ethiopia, 2017 (*n* = 511)

Variables	Categories	Frequency	Percent (%)
Age in years	< 20	111	21.7
	20–29	353	69.1
	30–39	47	9.2
Marital status	Married	483	94.5
	Single/divorced/widow	28	5.5
Maternal formal education	Yes	485	94.9
	No	26	5.1
The highest level of education	Primary school	159	31.1
	Secondary school	213	41.7
	Diploma and above	139	27.2
Occupational status	Employed	103	20.2
	Merchant	103	20.1
	Unemployed	305	59.7
Husband education	Yes	493	96.5
	No	18	3.5
Average monthly income	446–1200	73	14.3
	1201–2500	198	38.7
	2501–3500	95	18.6
	> 3501	145	28.4

### Psychosocial and clinical characteristics of respondents

Regarding social support, 183 (35.8%) and 301 (58.9%) of the participants had poor and moderate social support, respectively. Out of the participants, 84 (16.4%) had intimate partner violence, and nearly one-third (30.9%) had no autonomy in decision making. Of the respondents, 42 (8.2%) had mental illness in their first-degree relatives, only (*n* = 44) 8.6% had previous history of depression, and only 1.2% had chronic illness history (Table 2).

**Table 2:** Psychosocial and clinical factors of mothers attending maternal and child health clinics of the public health centers Bahir Dar, Northwest Ethiopia, 2017 (*n* = 511)

Variables	Categories	Frequency	Percent (%)
Social support	Poor	183	35.8
	Intermediate	301	58.9
	Good	27	5.3
Intimate partner violence screening	No	427	83.6
	Yes	84	16.4
Women autonomy/empowerment	No autonomy	200	39.1
	Has autonomy	311	60.9
Stressful life event screening	No stressful life events	384	75.1
	Has stressful life events	127	24.9
A family history of mental illness	Near family member	42	8.2
	Distant family member	64	12.5
	None	405	79.3
Previous history of depression	Yes	44	8.6
	No	467	91.4
Chronic illness history	Yes	6	1.2
	No	505	98.8
Chronic medication use	Yes	6	1.2
	No	505	98.8

### Obstetric characteristics of the respondents

Of all the respondents, 331 (64.8%) had their first pregnancy and nearly 1 out of 4 of the 133 (26%) women had hospitalized their babies. Nearly 1 out of 5 of the 88 (17.2%) women delivered by cesarean section and 89 (17.4%) had unintended pregnancy. Similarly, almost 3 out of 5 of the 326 (63.3%) respondents born female babies and only 25 (4.9%) did not desire for sex their last baby (Table 3).

**Table 3:** Obstetric factors of mothers attending maternal and child health clinics of the public health centers Bahir Dar, Northwest Ethiopia, 2017 (*n* = 511)

Variables	Categories	Frequency	Percent (%)
Number of pregnancies	1	331	64.8
	2–3	169	33.1
	> 4	11	2.2
Hospitalization of a baby	Yes	133	26
	No	378	74
Death of a child	Yes	11	2.2
	No	500	97.8
Mode of delivery	Vaginally	370	72.4
	C/section	88	17.2
	Instrumental	53	10.4
Planned pregnancy	Yes	422	82.6
	No	89	17.4
Sex of the last child	Male	185	36.2
	Female	326	63.8
Desired sex for the last child	Yes	486	95.1
	No	25	4.9

### The prevalence of postpartum depression

The 10 items of the Edinburgh Postnatal Depression Scale (EPDS) were summed to generate a single variable. The new variable ranges from 0 to 30 in absolute value. We further categorized the depression score into two levels (depressed and not depressed). A score greater than or equal to 13 indicated the existence of postpartum depressive symptoms. Nearly one in four (22.1%) with (95%, CI 18.6%, 25.8%) mothers had signs of postpartum depression.

### Factors associated with postpartum depression

Among all covariates, intimate partner violence, autonomous for decision making, stressful life events, hospitalized their baby, and unplanned pregnancy had less than 0.2 *P* value in the bivariable logistic regression and considered as the multivariate logistic regression models. The model goodness of fit was tested using the Hosmer and Lemeshow test, and a *P* value was found to be 0.56, which revealed as the model was good.

The multivariate analysis suggested that mothers with stressful life events were 4.4 times more likely to develop postpartum depressive symptoms compared to mothers who were not exposed to stressful life events (AOR = 4.4 (95%, CI 2.64,7.54). The odds of postpartum depressive symptoms increased by 4.2 times (95%, CI 2.54, 7.14) for mothers who did not have autonomy in the domestic decision making compared to their counterparts Unplanned pregnancy was about 1.9 times (95%, CI (1.02, 3.41) more risky for PPD compared to planned pregnancy.

Intimate partner violence was three times more likely to develop postpartum depressive symptoms than those who did not expose to intimate partner violence (AOR = 3.2; 95%, CI 1.76, 5.67). Those who had hospitalized their babies had 2.2 times (95%, CI 1.17; 4.31) risk of PPD compared to their counterparts (Table 4).

**Table 4:** Factors associated with postpartum depression among mothers attending maternal and child health clinics of the public health centers at Bahir Dar, Northwest Ethiopia, 2017 (*n* = 511)

Variables	Categories	Postpartum depression		COR (95% CI)	AOD (95% CI)
		Depression			
		No	Yes		
Stressful life events	No	333	52	1	1
	Yes	65	62	6.23 (3.95, 9.83)	4.46 (2.64, 7.54)***
Women autonomy in decision making	Yes	278	33	1	*1*
	No	120	80	5.62 (3.55, 8.8)	4.26 (2.54, 7.14)*
Hospitalization of a baby	No	312	66	1	1
	Yes	86	47	2.58 (1.66, 4.03)	2.24 (1.17, 4.31)*
Planned for pregnancy	Yes	339	83	1	*1*
	No	59	30	2.08 (1.26, 3.43)	1.86 (1.02, 3.41)**
Intimate partner violence	No	361	66	1	1
	Yes	37	47	6.95 (4.2, 11.51)	3.16 (1.76, 5.67)***
Previous history of depression	No	370	97	1	1
	Yes	28	16	2.18 (1.13, 4.2)	1 (0.40, 2.30)
Family history of mental illness	Near	22	20	3.7 (1.90, 7.10)	2.6 (0.14, 1.70)
	Distant	51	13	1.04 (0.54, 2)	0.7 (0.30, 1.60)
	No	325	80	1	1
Mode of delivery	Vaginally	290	80	1	1
	C/section	74	14	0.7 (0.40, 1.30)	0.67 (0.32, 1.40)
	Instrumental	34	19	2.03 (1.1, 3.70)	1.9 (0.90, 4)

* *P* value is significant at *P* < 0.05; ** *P* value is significant at *P* < 0.01

*** *P* value is significant at *P* < 0.001; 1 = reference

*P* value of Hosmer and Lemeshow test = 0.56

## Discussion

This study revealed that the prevalence of postpartum depressive symptoms was found to be 22.1% (95%, CI 18.6%, 25.8%). Regarding prevalence, the current study result is in line those of others studies carried out in Ethiopia, Kenya, Nigeria, and Pakistan, with the prevalence estimated at 19%, 20%, 22.9%, and 22.3%, respectively [5, 15, 32, 33].

On the other hand, our findings are higher than those of other studies done in three areas of Ethiopia, Sudan, Kenya, Egypt, Ghana, and the USA, where the prevalence was estimated at 12.2%, 13.11%, 16.3%, 9.2%, 13%, 7.14%, 7%, and 14.8%, respectively [4, 8, 34–39]. The variation may be due to distinctions in sample sizes, measurement tools, rating scales, timing of postpartum period, study designs, and socio-cultural differences between Ethiopia and the other countries. In South Ethiopia, 3147 participants were included between 1 and 12 months of the postpartum period by using Patient Health Questionnaire nine [34]. Only 196 participants were included in the northern part of Ethiopia by assessing Beck Depression Inventory Scale (BDIS) with the cutoff points greater than 14 [36] and in East Ethiopia, 122 postnatal mothers had participated by using the non-probability convenience sampling technique [35]. In Sudan, 238 pregnant women took 3 months with PPD, but in this study included from 2 to 6 weeks of postpartum period by using EPDS at the cutoff greater or equal to 12 [8]. In Kenya, 200 participants from 6 to 14 weeks duration were included [37]. In Egypt, 658 women took part within 6 weeks after birth [4], and in Ghana, only 212 mothers selected by using convince sampling methods used to assess by Patient Health Questionnaire (PHQ-9) [38]. Finally, in the USA, 325 mothers from community health centers using the Pregnancy Risk Assessment Monitoring System (PRAMS) and stratified sampling techniques were included [39].

The result of the study was lower than those of other studies conducted in South Africa, China, Turkey, and Bangladesh, with the prevalence estimated at 50.3%, 30%, 35.2%, respectively [40–42]. This might be due to measurement tools, sample sizes, and duration. For instance, in South Africa, 159 participants were included from the rural community within 4–14 weeks of the postpartum period using a cohort study design [40]. In Bangladesh, EPDS was used with the cutoff point 10 or more to define clinically significant symptoms of postpartum depressive symptoms [42]. In China, 506 participants were assessed using the Center for Epidemiologic Studies Depression Scale (CES-D) to measure participants’ depression symptoms with the cutoff point greater than or equal to 16 and to detect people at risk of experiencing depressive disorder on mothers 1–3 years of the postpartum period [41].

Intimate partner violence was three times higher compared to mothers who did not expose to intimate partner violence. Partner violence during pregnancy was significantly associated with depressive symptoms in the rural area of Ethiopia [43]. A study was done in Sudan, in which the history of domestic violence increased seven times the postnatal depression [8]. In a cross-sectional study in four sub-Saharan African countries, intimate partner violence was the risk factor for developing depressive symptoms [44]. In South Africa, intimate partner violence during pregnancy and postpartum period had a risk of depressive symptom severity [45]. In China, violence was one of the factors responsible for postpartum depressive symptoms [46]. A study was done in the UK on domestic violence among women with a high level of depressive symptoms in the antenatal and postnatal periods [47]. In the USA, the rates of mental health problems were at least three to five times higher in women exposed to intimate partner violence [48]. In another meta-analysis study done in India, the presence of domestic violence increased postnatal depressive symptoms [49]. The explanations for the association may be that violence could lead to psychological trauma; especially if the action was in front of other people, neighbors, friends, and relatives, psychological trauma leads to a negative belief about self and others.

The odds of postpartum depression were increased by two times among mothers who had no autonomy in domestic decision making than those who had autonomy. Despite the variability and broadness of the concept of autonomy in this study, women empowerment typically refers to freedom of movement, decision making regarding children, and decision making in household tasks. This finding is supported by those of other studies in Nepal and Bangladesh [12, 42]. A study in China showed that low autonomy to decide indicated a high chance to develop mental health problem [50]. The explanation for this could be that if women had limited right and control over resources, restriction in their mobility, lack of personal power in intra-household ability to make and execute independent decisions based on her own concern or about children, this inequality could result in some psychological traits that negatively affect her cognition.

This study found that postpartum depressive symptoms were 2.8 times higher among respondents who experienced stressful life events compared to mothers who did not experience these. The finding in Egypt supported this result, and a history of different psychosocial stressors was a predictor of postpartum depressive symptoms [7]. In the UK, events related to the partner relationship during pregnancy and postpartum were risk for depression onset, especially the occurrence of related events in the preceding months [11]. A study was done in the USA, which showed that different life stressors were a factor to develop postpartum depressive symptoms [51].

In contrast, in a study on psychosocial risk and protective factors for postpartum depressive symptoms in the United Arab Emirates, neither anxiety nor depression and a number of stressful life events were found to be risk factors [9]. The explanation for the association could be stressful life events acting as the onset persistence of depression. Mothers who had hospitalized their babies were found to be 2.7 times more likely to become depressed than their counterparts. This finding is consistent with those of other studies carried out in Egypt [7], and India [52] in that puerperal woman had the ill baby become more stressed about the health outcome of their infant.

Unplanned pregnancy was about two times risky for PPD compared to planned pregnancy. This finding is in line with those of other studies carried out in Southern Ethiopia. Unwanted pregnancy was the risk factor for depressive symptoms among pregnant women living in rural area [43]. In Egypt, unwanted pregnancy was the predictor of postpartum depressive symptoms [7]. In India, those who had mood swings during pregnancy and those who had low mood during pregnancy [53] and in China women with cesarean delivery and unplanned pregnancy had risk factor for postpartum depressive symptoms. In the USA, unplanned pregnancy was a predictor of postpartum depressive symptoms [50, 51].

The explanation for the association might that unwanted pregnancy causes psychological distress in women. Unplanned pregnancy could lead to negative emotional reaction and increase psychosocial stress, decrease support provided to her by the partner, and risk of having depression. This study was a cross-sectional design cannot permit conclusions for some variables, for example, to decide whether PPD are risks for or consequence. The finding is likely only to hint at the complex interactions between PDD and explanatory variables. Another limitation was that we did not use a standardized screening tool to assess women’s autonomy. Therefore, the interpretation and usage of the study must be considered a limitation.

## Conclusions

In this study, the prevalence of postpartum depressive symptoms (22.1%) was high. Stressful life events, lack of empowerment in domestic decision making, intimate partner violence, unplanned pregnancy, and hospitalization of their baby were factors significantly associated with postpartum depression. The Ministry of Health should develop guidelines to screen and treat postpartum depressive symptoms among mothers attending the MCH service. Further research on risk factors of postpartum depressive symptoms should be conducted to strengthen and broaden these findings.

## Data Availability

The dataset during and/or analyzed during the current study available from the corresponding author on reasonable request.
